# Relationship between periodontal diseases and newly-diagnosed metabolic syndrome components in a sub-Saharan population: a cross sectional study

**DOI:** 10.1186/s12903-021-01661-6

**Published:** 2021-06-29

**Authors:** Jean Xavier Ekouelkoum Ngoude, Vicky Jocelyne Ama Moor, Tsobgny Tsague Nadia-Flore, Batakeh B. Agoons, Gamgne Guiadem Catherine Marcelle, Elage Epie MacBrain, Daryl Nzokou Tcheutchoua, Jan René Nkeck

**Affiliations:** 1 Faculty of Medicine and Biomedical Sciences, University of Yaoundé I, Yaoundé, Cameroon; 2 Biochemistry Laboratory, University Teaching Hospital of Yaoundé, Yaoundé, Cameroon

**Keywords:** Periodontal diseases, Metabolic syndrome, Public health, Cameroon

## Abstract

**Background:**

Oral health is a frequently ignored aspect of global health in sub-Saharan patients. Periodontitis, a very frequent oral disease has been proven to be associated to development of the metabolic syndrome. This study aims to evaluate the relationship between periodontal disease and metabolic syndrome components in a sub-Saharan population.

**Methods:**

We performed a cross sectional study in 3 Yaounde hospitals. Consenting adults aged 21 years and above were recruited. Participants who presented with a tooth loss of at least 50% or any condition which could alter values of biological and periodontal parameters (tobacco smoking, pregnancy, chronic kidney disease, cancer) were excluded. Metabolic syndrome elements (glycaemia, arterial pressure, HDL cholesterol, abdominal circumference, triglycerides) and periodontal variables were recorded (plaque and gingival index of Silness and Loe, periodontal pocket depth and clinical attachment loss). These variables were compared using Fisher’s exact Test and odds ratio calculated with 95% confidence intervals.

**Results:**

The prevalence of periodontitis and metabolic syndrome were 43.4% and 10.8% respectively. Age (37.75 ± 13.25, *P* < 0.001) and poor accessory brushing methods were associated risk factors for development of periodontal disease. Sub-Saharan sindividuals with periodontitis had increased odds of having obesity (OR 11.1 [95% CI 3.97–31.03], *P* < 0.001) and low HDL (OR 4.58 [95% CI 1.79–11.70], *P* = 0.001)

**Conclusion:**

Our findings suggest an association between periodontal disease and metabolic syndrome in Sub-Saharan subjects. Increasing age and poor accessory brushing methods are associated risk indicators.

## Background

Metabolic syndrome is a complex and multifactorial disorder, incorporating both clinical and biological disturbances [1]. These conditions include abdominal obesity, dyslipidemia, hyperglycemia and hypertension, and arise from insulin resistance and impaired adipose deposition and function. Each component of the metabolic syndrome is a modifiable cardiovascular risk factor, and the combination of these factors leads to an increase in overall cardiovascular risk, making the metabolic syndrome a major public health problem [2, 3]. As such, efficient control of each component is critical in order to decrease mortality [4].

Oral health status is usually ignored when global health status of an individual is taken under consideration [5]. Interest in the effect of oral health on the prevention of systemic disease has gained importance in recent years with researchers recognizing the bi-directional relationship between both of them [6]. Periodontitis, one of the most frequent oral diseases, is defined as an inflammation of the supporting structures of the teeth, with a progressively destructive change eventually causing loss of bone and periodontal ligament [7]. It is caused by a multifactorial imbalance between the microbial flora and the host’s defense mechanisms [8].

Recent epidemiological studies report an expansion of non-communicable diseases in sub-Saharan Africa, namely hypertension, diabetes, coronary heart disease [9]. The common feature of these diseases is the metabolic syndrome, which is an entity at the basis of any cardiovascular risk equation, and is a major contributor to morbidity and mortality in our environment according to the World Health Organization [10]. The frequency of periodontal diseases is also increasing worldwide in parallel with the frequency of metabolic syndrome [11]. This study attempts to explore the associated factors to periodontal disease and the metabolic syndrome components in Cameroon, as a way of helping health professionals to prevent cardiovascular risk by promoting good oral health.

## Methods

### Ethical considerations

The study protocol was approved by the Centre Regional Ethics Committee for Research in Human Health, Cameroon (3435/CRERSHC/2020). All study procedure was done in accordance with the 2013 revised Helsinki Declaration and the Good Clinical Practice guidelines of the International Conference on Harmonisation. All participants provided a written informed consent form before enrollment into the study.

### Study population and design

A cross sectional study was performed from January 2020 to May 2020 in three hospitals of Cameroon. Participants for this study were pooled from a population of Cameroonian adults consulting at two tertiary hospitals in Yaoundé, Cameroon. The study design was a cross-sectional one, and ran for a period of 4 months (January 13, 2020 to May 14, 2020). We included in this survey all adults aged 21 years and above, who gave a written consent to participate. Subjects with chronic diseases (diabetes, chronic kidney disease, hypertension, cancer), dyslipidemia, history of tobacco consumption, pregnant women or women taking contraceptives, and subjects with a tooth loss greater than 50% were excluded.

Accruing evidence from various studies prove the harmful effect of oral contraceptives [12, 13] and tobacco consumption [14, 15] on periodontal health. Subjects with already diagnosed high blood pressure, diabetes or dyslipidemia were excluded from this study in order to eliminate conditions capable of increasing the prevalence of periodontal disease and/or metabolic syndrome. The community index used to assess periodontal disease was based on 20 teeth. As such, subjects with 50% tooth loss (16 teeth) were excluded. Also, those with significant tooth loss (50% and more) were not enrolled because of the uncertainty of the etiology of the tooth loss.

The Cochran formula [16] was used to calculate the minimum number of subjects required for this study;

$${\mathbf{n}}\, = \,{\mathbf{Z}}^{{\mathbf{2}}} \frac{{\user2{p}\left( {1 - \user2{p}} \right)}}{{\user2{e}^{2} }}$$

where n = sample size, Z = standardized normal deviate (1.96), p = prevalence of metabolic syndrome, e = precision.

Prevalence of metabolic syndrome was gotten from the Brazilian study of Gomes-Filho et al. [17] (*p* = 67.1% according IDF criteria for Metabolic syndrome definition) and the values for other variables obtained from statistic tables. Therefore, our minimum sample size was 84 participants.

### Data collection

After collecting data on lifestyle habits, we performed a complete clinical examination, documenting weight, height, waist circumference and blood pressure. Periodontal examination for periodontal parameters assessment was then performed. Blood samples were collected in order to assess glycaemia, HDL-cholesterol and triglycerides.

### Periodontal examination

Each participant underwent a complete oral examination performed by a certified periodontist using a dental mirror and a manual periodontal probe (graduation 1–15 mm, *Dentsply maillefer®)*. The oral cavity was separated into six sextants (17–14, 13–23, 24–27, 37–34, 33–43, 44–47) and the Silness and Loe plaque index, probing depth (PD), gingival index, clinical attachment loss (CAL) were evaluated on the maxillary, mandibular, first and second molars, the maxillary right central incisor and the mandibular left central incisor teeth [18].

According to the criteria of the American academy of Periodontology; Gingivitis was diagnosed if a gingival index score of 1 was present on two non-adjacent teeth. Periodontitis was diagnosed as localized or generalized, and differentiated on the basis of clinical attachment loss and pocket depth, into Mild (CAL; 1–2 mm, PD 3–4 mm), Moderate (CAL; 3–4 mm, PD 5–6 mm), and Severe (CAL; 5 mm, PD ≥ 7 mm), on at least 2 non-adjacent teeth [19].

### Assessment of the metabolic syndrome

Trained study personnel measured blood pressure twice in a seated position with an automated sphygmomanometer (AND UA 767S®, Kyoto, Japan) and an appropriately adult sized cuff according to a standard protocol after at least 5 min of rest prior to the initial blood pressure reading. Systolic (SBP) and diastolic blood pressure (DBP) values were taken as the average of two measurements recorded > 2 min apart. At the baseline examination, after a fasting period of 12 h, blood from each participant was sampled, and dosage for triglycerides, high-density lipoprotein (HDL) cholesterol, and fasting blood glucose done. The metabolic syndrome was defined using the International Diabetes Federation Criteria [20];

1. abdominal obesity, was diagnosed if waist circumference of ≥ 94 cm for males and ≥ 80 cm (for females).
2. Hypertriglyceridemia (serum triglyceride > 150 mg/dL).
3. Low HDL-cholesterol (< 40 mg/dL for males and < 50 mg/dL for females or specific treatment for this lipid abnormality).
4. High blood pressure (systolic ≥ 130 mmHg and diastolic ≥ 85 mmHg).
5. Fasting serum glucose (≥ 100 mg/dL).

The diagnosis of metabolic syndrome was retained when abdominal obesity was present with at least two of the above abnormalities.

### Lifestyle variables

Gender, age, frequency of daily toothbrushing, education level, and oral hygiene practices (usage of complementary brushing methods) were documented. The frequency of daily toothbrushing was evaluated as the number of tooth brushings performed the previous day.

### Statistical analysis

Data were analyzed using S.P.S.S (Statistical Package for Social Sciences) version 23.0 software for statistical analysis. Results were presented as means and standard deviation and counts with percentage. The graphs were created using Microsoft® Office Excel 2013 and S.P.S.S. version 23.0. After testing for normality, the student’s *T* test was used to compare the means between two quantitative variables. Proportions were compared using the Fisher’s exact test. The odds ratio (OR) and its 95% confidence interval were used to quantify the degree of association between qualitative variables. For all the tests used, the significance threshold was set at 0.05.

## Results

A total of 149 subjects were invited to take part in this study, with 83 finally enrolled. 30 participants with type 2 diabetes, 13 subjects with a positive psychostimulant consumption history, 14 participants with documented hypertension and 9 women who presented a positive urinary pregnancy test were excluded.

### Sociodemographic and lifestyle characteristics

In the study population with a mean age of 32.3 ± 12.1 years, 43 (51.8%) were women. Seventy-five percent (n = 62) of our population had a history of dental consultations, with toothache being the primary symptom (39.8%). Oral health history of the participants as shown in Table 1.

**Table 1:** Oral health history of the participants

Variables; n (%)	Number (N = 83)	Percentage (%)
Dental consultation		
Yes	62	74.7
Tooth brushing frequency/day		
Once	27	32.5
Twice	54	65.1
≥ Thrice	02	2.4
Tooth brushing period (with respect to meals)		
Before	19	22.9
After	16	19.3
Before and after	27	32.5
Either before or after	21	25.3
Usage of accessory brushing methods		
Yes	32	38.6
Accessory brushing methods		
Toothpicks	09	10.8
Inter dental brush	03	3.6
Mouth wash	10	12.0
Dental floss	10	12.0
None	51	61.4

### Periodontal disease indices and associated factors

The prevalence of periodontal disease was 43.4% (n = 36) in our population. Mild periodontitis was the most represented form of periodontal disease (41.7%, n = 15). The periodontal indices revealed that about 3⁄4 (73.5%) of our population had no clinical loss of attachment and only 26.5% (n = 22) had a pocket depth higher than 3 mm. Bacterial plaque visible to the naked eye was present, and had a mean plaque index of 1.81 ± 0.56 and a mean gingival index of 0.57 ± 0.74.

Results from our study population showed that increasing age (37.75 vs 28.0, *p* < 0.001), and the absence of accessory brushing methods were significantly associated with the development of periodontal disease (09 vs 23, *p* = 0.026). Comparisons of other associated factors revealed no statistically significant difference, as shown in Table 2.

**Table 2:** Factors associated with periodontal disease

Variables	Presence of Periodontal disease	Absence of Periodontal disease	*P* value
Age (μ ± SD)*	37.75 ± 13.25	28.0 ± 9.36	< 0.001
Gender n (%)			
Male	15 (18.1)	25 (30.1)	0.298
Number of dental consultations n (%)			
Yes	24 (28.9)	38 (45.5)	0.141
Tooth brushing frequency/day n (%)			
Once	11 (13.3)	16 (19.3)	0.440
Twice	23 (27.7)	31 (37.3)	
≥ Thrice	02 (2.4)	0 (0)	
Accessory brushing methods			
Yes	09 (10.8)	23 (27.7)	**0.026**

Bold value indicate p values < 0.005

### Metabolic syndrome

**Fig. 1:**
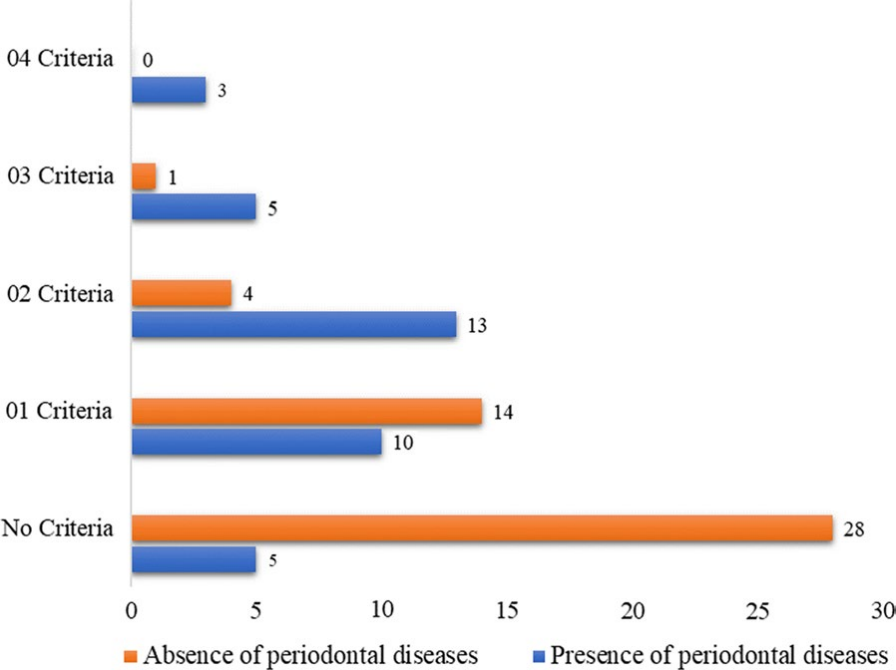
Distribution of periodontal diseases according to Metabolic syndrome criteria

Nine over the total 83 participants had 3 or more components of the metabolic syndrome, giving a prevalence of 10.8%. Among the periodontal disease-free patients, 50.6% had at most 01 element of the metabolic syndrome. Graphical distribution of the elements of the metabolic syndrome with respect to the presence and absence of periodontal disease is shown in Fig. 1.

### Association between periodontal disease and metabolic syndrome

The difference in the number of metabolic syndrome criteria was statistically significantly higher (*p* = 0.001) in patients with versus without periodontal disease.

Having periodontal disease was associated with high levels of triglycerides (3 vs 0, *p* = 0.044), abdominal obesity (27 vs 10, *p* = 0.001) and high blood pressure (7 vs 1, *p* = 0.008) (This is represented inTable 3).

**Table 3:** Characteristics of the metabolic syndrome and its components

Variables; n (%)	Presence of Periodontal diseases (n = 36)	Absence of Periodontal diseases (n = 47)	*P* value	OR [95%CI]
Components of the metabolic syndrome				
Abdominal obesity	27 (73.0)	10 (27.0)	**< 0.001**	**11.1 [3.97–31.03]**
High blood pressure	7 (87.5)	1 (12.5)	**0.008**	**11.1 [1.29–94.96]**
Low HDL-C	22 (64.7)	12 (35.3)	**0.001**	**4.58 [1.79–11.70]**
Triglycerides	3 (100)	0 (0.0)	**0.044**	**–**
Fasting blood sugar	3 (60)	2 (40)	0.439	–
Metabolic syndrome	8 (9.6)	1 (1.2)	**0.004**	**13.14 [1.56–110.37]**
Number of criteria				
01	10 (12)	14 (16.9)	** < 0.001**	–
02	13 (15.7)	4 (4.8)		
03	5 (6.0)	1 (1.2)		
04	3 (3.6)	0 (0)		

Bold value indicate p values < 0.005

## Discussion

In this cross-sectional study of a group of sub-Saharan individuals, we sought to assess the relationship between periodontal diseases and newly diagnosed metabolic syndrome. Results showed a periodontitis prevalence of 43.4%, and a Metabolic syndrome prevalence of 10.8%. After odds analysis, we found that subjects with periodontal disease had increased odds of having metabolic syndrome, and were more likely to have co existing abdominal obesity and low HDL levels.

Our findings are similar to those of previous studies evaluating periodontitis and metabolic syndrome [11, 21–25]. Studies evaluating the prevalence of periodontal disease in the same geographical area have been conflicting, with a 2017 community study [26] estimating periodontal disease prevalence at 32.1%, and another calculating metabolic syndrome prevalence at 5.9% in 2007. Wu et al. [27] showed the effect of increasing age on periodontitis, with Franceschi et al. [28] documenting periodontitis as a possible occurrence on the increasing age spectrum. Advancing age is a complex multifactorial process that increases susceptibility to chronic inflammatory diseases and microbial infection. Older individuals tend to have higher levels of gram-negative germs which colonize oral mucosa. These collectively lead to periodontal tissue damage, and subsequent periodontitis [8, 29]. Poor dental hygiene, as evidenced by a lack of accessory brushing methods, remains the foundation for periodontal tissue damage [30]. This is supported by this study showing poor brushing methods being a risk indicator for periodontal damage.

Metabolic syndrome was diagnosed in 9 participants who represent 10.8% of our study sample. A previous study about metabolic syndrome carried out by Ntentie and al in a sample of 5654 volunteers reported a prevalence of 12.7% in urban areas and 7% in rural areas. This emphasizes the thought that the increase in metabolic syndrome prevalence is due to the progressive urbanization of the city these participants live in, with increasing diet based on unhealthy foods and drinks [31]. Yaounde, the capital city of Cameroon, is home to an estimated 2.8 million citizens, and has undergone rapid urbanization and modern development in recent years [32]. Our results showed that 8 of the participants with metabolic syndrome also presented periodontal disease, be it gingivitis, mild or moderate periodontitis. No case of severe periodontitis was found, probably because of the reduced mean age (32.3 ± 12.1 years) and the exclusion of participants presenting factors which affect periodontal disease expression like tobacco smoking and pregnancy. The IDF criteria for metabolic syndrome diagnosis contain at least 3 components which increase patients’ susceptibility to periodontal disease: hyperglycemia, obesity and dyslipidemia.

The purpose of this study was to investigate the relationship between periodontal disease and metabolic syndrome. By using the Fisher’s Extract test, we found sub-Saharan individuals with periodontal disease had increased odds of having metabolic syndrome.

When analyzing the association between periodontitis and metabolic syndrome, other surveys reported two types of association: an association where periodontitis is exacerbated in cases of metabolic syndrome and an association where metabolic syndrome occurrence is increased in patients with periodontitis. This highlights a two-way relationship between both pathologies and can be explained by a cause-effect relationship underlined by chronic low-grade inflammation common to periodontal disease and the components of metabolic syndrome we cited above. Inflammatory markers involved in metabolic syndrome (TNF-α, IL-1, IL-6, PAI-1) can upregulate periodontal disease when a persistent periodontal inflammation can exacerbate systemic inflammation, insulin resistance and endothelial dysfunction [33].

Abdominal obesity, high blood pressure, low HDL-C and triglycerides were found to be frequent in participants with periodontitis. This supports the previous findings of Morita and al in 2009, Miki and al and Lamster and al in 2017. As Shimazaki and al in 2007 and Morita and al in 2009, we also find that increase gingival index and periodontal pocket depth was associated with increase triglycerides levels and low HDL-C. During periodontal disease hyperactivation of neutrophils and expression of proinflammatory cytokines (adipokines, TNF-α, IL-1, IL-6, CRP), oxidative stress leads to insulin resistance with disturbance of carbohydrates, lipids and proteins metabolism and therefore to obesity, hypertension and hyperglycemia [22, 23, 34, 35].

The present study has some limitations. It was a cross sectional study designed to explore the relationship between periodontal disease and metabolic syndrome, and is therefore not enough to explore rigorously causal relationship between the conditions of interest. Secondly, we did not analyze the prevalence of metabolic syndrome according to periodontal disease severity. Therefore, large scale longitudinal studies ought to be carried out in order to clearly characterize this association. In addition, exclusion of subjects with diabetes and hypertension, due to the effect of these conditions on periodontal health, possibly underestimates the prevalence of the metabolic syndrome. Also, the authors obtained odds ratio values, as opposed to prevalence ratios, which are more accurate. This raises the possibility of results overestimation. Nevertheless, the results presented in this study present a certain clinical significance in sub-Saharan Africa settings and exhibit an interest on a public health scale.

Our findings provide data to guide clinical practice of health professionals in Cameroon, and sub-Saharan Africa, and to guide future research. In a region experiencing a rise in metabolic syndrome prevalence, the results of this study seek to draw attention to the fact that early diagnosis and accurate treatment of periodontitis may greatly reduce the odds of developing metabolic syndrome. Development of public health campaigns with the aim of sensitizing the public to the importance of good dental hygiene, accessory brushing methods, techniques and frequency of brushing will be important in the overall control of cardiovascular risk.

## Conclusion

This study examined the relationship between periodontitis and metabolic syndrome, and investigated the associated factors related to their development. The results suggest that periodontal diseases and the metabolic syndrome are linked. Age and toothbrushing patterns being associated with periodontal disease, oral health professionals in sub-Saharan areas should recognize the importance of periodontal health, in reducing the incidence of metabolic risk, and protection against future cardiovascular events.

## Clinical relevance

*Scientific rationale for study* The relationship between periodontal disease and metabolic syndrome in Cameroonian and sub-Saharan African subjects have been under-reported, due to a lack of concrete data. As such, the importance of boosting global health through the promotion of oral health is frequently ignored.

*Principal findings* In this study, we report on the prevalence of periodontal disease and metabolic syndrome in this population. We also document the strength of their association, and determine risk factors for periodontal disease.

*Practical implications* Our results show what aspects of oral hygiene health professionals should insist on, to reduce risk of periodontal disease.

## Data Availability

The datasets generated and/or analyzed during the current study are not publicly available (due to the absence of an adequate privacy-ensuring public repository in the country). However they are available from the corresponding author on reasonable request.
